# Surface Enhanced Raman Scattering in Graphene Quantum Dots Grown via Electrochemical Process

**DOI:** 10.3390/molecules26185484

**Published:** 2021-09-09

**Authors:** Rangsan Panyathip, Sukrit Sucharitakul, Surachet Phaduangdhitidhada, Athipong Ngamjarurojana, Pisist Kumnorkaew, Supab Choopun

**Affiliations:** 1 Center of Excellence in Physics and Astronomy, Department of Physics and Materials Science, Faculty of Science, Graduate School, Chiang Mai University, Chiang Mai 50200, Thailand; rangsanpanyatip@gmail.com (R.P.); sukrit.sucharitakul@cmu.ac.th (S.S.); surelity@gmail.com (S.P.); ngamjarurojana@yahoo.com (A.N.); 2 National Nanotechnology Center (NANOTEC), National Science and Technology Development Agency, Pathumthani 12120, Thailand; pisist@nanotec.or.th

**Keywords:** graphene, quantum dots, surface enhanced Raman, electrochemical process, electrolyte

## Abstract

Graphene Quantum dots (GQDs) are used as a surface-enhanced Raman substrate for detecting target molecules with large specific surface areas and more accessible edges to enhance the signal of target molecules. The electrochemical process is used to synthesize GQDs in the solution-based process from which the SERS signals were obtained from GQDs Raman spectra. In this work, GQDs were grown via the electrochemical process with citric acid and potassium chloride (KCl) electrolyte solution to obtain GQDs in a colloidal solution-based format. Then, GQDs were characterized by transmission electron microscope (TEM), Fourier-transform infrared spectroscopy (FTIR), and Raman spectroscopy, respectively. From the results, SERS signals had observed via GQDs spectra through the Raman spectra at D (1326 cm^−1^) and G (1584 cm^−1^), in which D intensity is defined as the presence of defects on GQDs and G is the sp^2^ orbital of carbon signal. The increasing concentration of KCl in the electrolyte solution for 0.15M to 0.60M demonstrated the increment of Raman intensity at the D peak of GQDs up to 100 over the D peak of graphite. This result reveals the potential feasibility of GQDs as SERS applications compared to graphite signals.

## 1. Introduction

Surface-enhanced Raman spectroscopy (SERS) is considered to be a label-free and ultra-sensitive detection of biological molecules and chemical species by optical techniques. It is a powerful technique used in characterizing the structures of materials based on resonant Raman scattering. The development of SERS techniques for enhancing Raman signals has made Raman spectroscopy more popular in many kinds of research and applications. SERS enhancement factor can be computed by comparing the signal intensity of the normal Raman spectroscopy on the surface with that of a modified surface of the substance of interest. However, identification of the SERS enhancement factor can be difficult [1] due to the usual signal-to-noise ratio of normal Raman.

SERS is composed of two widely accepted mechanisms: An electromagnetic mechanism (EM) and a chemical mechanism (CM). SERS in the EM mode is based on the local electromagnetic field that is contributed to by the electromagnetic enhancement of the surface plasmons generated upon light irradiation [2,3,4,5]. The EM model is often set up using a metal ion coated on the substrate with proper micro-nano scaled geometries. With nano-scaled metal, surface plasmon resonance signals in this mode can be hosted resulting in the strong SERS signals for up to 10^10^ times of normal signals [6]. However, SERS in the EM mode requires complex approaches in preparing nanostructures on the substrate for SERS given optimized conditions. In contrast, the CM mode is the SERS mechanism, which depends on the chemical bonding of molecules absorbed by the substrate, which is described by free-electron charge transfer between molecules bonding, inducing a 10–100-fold enhancement of surface signals in this model [7,8]. However, SERS signals observed often include EM and CM modes as a coupling process, in which the EM mode would show a level of detection higher than the CM mode due to the strong signals of the hot spot by surface plasmons between two metal particles generating the high intensity of SERS signals [1]. As the CM mode utilizes only the molecules directly in contact with the substrate for inducing SERS by the influence of charge transfer between the molecule and the substrate, SERs in such a mode does not require complicated metal nanostructures, and allow the use of biocompatible materials as the source [9]. Therefore, SERS in the CM mode is often used to collect SERS information for wide applications, for example, the biosensor for cancer cell detection, drug tracking, and other high-sensitivity molecule detection applications [10,11].

It has been reported that graphene can be used as a SERS substrate material for detection in the CM mode [9]. Graphene debuted as a 2D structure [12] by the in-plane monolayer of carbon atoms in a honeycomb-like structure and has an aromatic carbons structure, exposed hydrophobic properties, and is inert for a chemical reaction [13]. Furthermore, graphene does not support the EM mode but requires rough surface modification with metal particles for producing surface plasmons in the EM mode. Recently, GQDs have become the carbon material structure, like graphene, in a 0D species that displayed features of fluorescence and have applications om optical sensing, bio-medical, optoelectronic devices, photo-science, bio-imaging, and SERS detection [14]. SERS in GQDs can be extremely useful as the material not only features compatibility with the SERS mechanism and environmental friendliness, but also biocompatibility, high chemical stability, and large specific surface areas [13], which supports the enhancement process of the SERS mechanism. Recently, SERS in GQDs has been reported by D. Liu [15], who synthesized GQDs via plasma chemical vapor deposition (P-GQDs) for sizes of 2.2 nm. Then, they treated P-GQDs by rhodamine molecules at a concentration of 10^−9^ M on the P-GQDs surface. As a result, the SERS signals were observed on their P-GQDs spectra, which are explained as the SERS in the CM mode. In addition, the SERS in their work was explained via electron charge transfer between hydrogen bonding of P-GQDs edges and hydroxyl molecules of rhodamine, of which both molecules had matching Fermi level energy. Then, R. Das and coworkers [16] also reported SERS in the n-type GQDs semiconductors (N-GQDs), in which N-GQDs were synthesized from graphene oxide by Hummer’s method and the solvothermal method to achieve N-GQDs in dimethylformamide solvent (3 nm of size). SERS on N-GQDs has a high sensitivity for rhodamine detection at a low concentration for 10^−10^ M by using the mechanism of the charge transfer process between N-GQDs and rhodamine molecules.

However, all the previous works reported the SERS on GQDs in solid form, which may require a complicated process in order to apply GQDs in solid form for use in other SERS applications when compared with GQDs in the solution-based format. In addition, solution-based GQDs could be synthesized via the top-down approach, such as an electrochemical method, which allows the possibility to obtain SERS from GQDs as solution-based. This method used the electric field to drive the chemical reaction and have the process of molecules contact, which is the mechanism for support SERS on the CM mode. Although solution-based GQDs are of interest to study, S. Ahirwar and coworkers reported the synthesis of GQDs through the electrochemical exfoliation method from graphite rods as carbon source electrodes in citric acid and a NaOH mixing electrolyte solution. Interestingly, they reported the hydroxyl ions (OH^−^) in the electrolyte served for the extraction of nanoscale GQDs out of graphite electrodes, but they did not find SERS from their GQDs results [17]. This might be due to the weak signals of SERS on GQDs often combined with the strong noise signals, which induce difficulty in distinguishing SERS signals from typical Raman signals. Therefore, it is important to understand each mechanism process that contributed to the SERS appearing on GQDs and to explain the process of the SERS enhancement on a solution basis.

In this work, GQDs are prepared by the electrochemical reaction process of electrolytes in solution at a constant of citric acid (0.1M) and various KCl concentrations for 0.15M–0.60M and are characterized and investigated. SERS signals are observed and analyzed. The enhancement of Raman signals is discussed and explained by the mechanism of the CM mode.

## 2. Results and Discussions

### 2.1. Synthesis of GQDs via an Electrochemical Process

**Figure 1:**
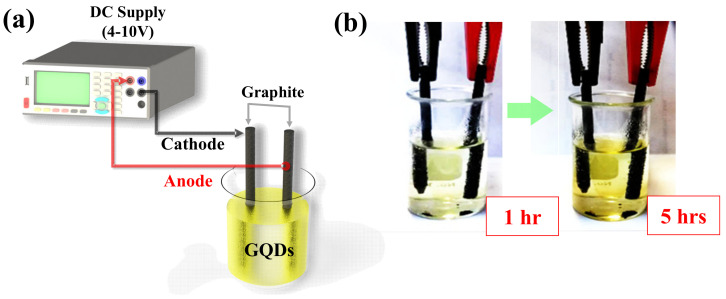
(**a**) Schematic setup of GQDs synthesis in the solution-based form via the electrochemical process and (**b**) progress of GQDs synthesis at 1 h to the finished process of 5 h.

GQDs were grown by the electrochemical method as illustrated in Figure 1a utilizing bare graphite rods as precursors. The numerous defects on the bare graphite rod surface are produced by the annealing process at 450 °C for 15 min [17] to increase the etching and oxidation rate on the graphite surface. Figure 1b shows the progress of the electrochemical exfoliation process for synthesis GQDs at hour 1 and hour 5 of the synthesis, respectively.

It can be seen that the solution color changes to yellow and becomes darker after 5 h indicating a higher concentration of GQDs at a longer process time. GQDs are displayed as the clear yellow color solution for all growth conditions. Tyndall effect examination checked solution-based GQDs through a typical red laser pointer, which observed the red laser light path in all solutions, that is, confirming GQDs as fine nanoparticles in a colloidal solution.

### 2.2. Characterizations of GQDs

**Figure 2:**
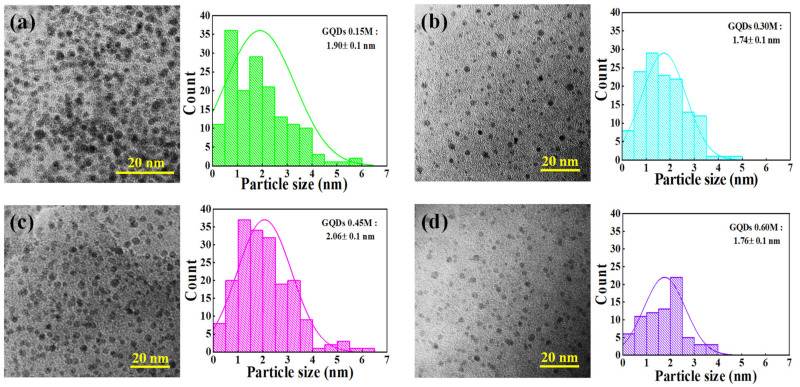
TEM images of GQDs with the particle size distribution for electrolyte concentrations of (**a**) 0.15M, (**b**) 0.30M, (**c**) 0.45M, and (**d**) 0.60M, respectively.

GQDs were then analyzed to identify the morphology via TEM images, as shown in Figure 2. From TEM images, typical GQDs particles are observed in a spherical shape with a similar diameter on each condition at a range of 1.7 to 2.0 nm. The sizes of GQDs are systematically analyzed by ImageJ software [18,19] with the distribution curve on the left of TEM images that separated each condition of GQDs (0.15M–0.60M) in the synthesis process. It should be noted that the TEM images confirmed all growth conditions in the electrochemical exfoliation process and each condition in the electrolyte achieved GQDs.

**Figure 3:**
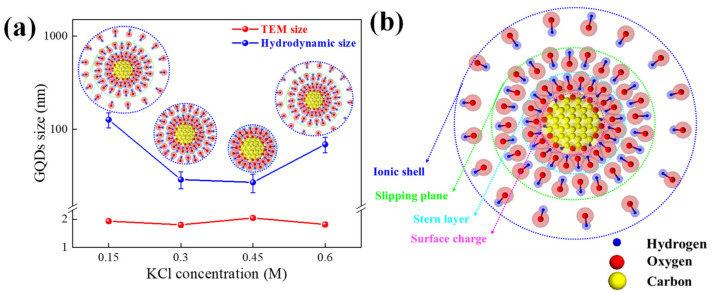
(**a**) GQDs size of the hydrodynamic size and TEM size at various electrolyte concentrations and (**b**) hydrodynamic size model of GQDs including the surface charge layer, stern layer, slipping plan layer, and ionic shell layer.

**Figure 4:**
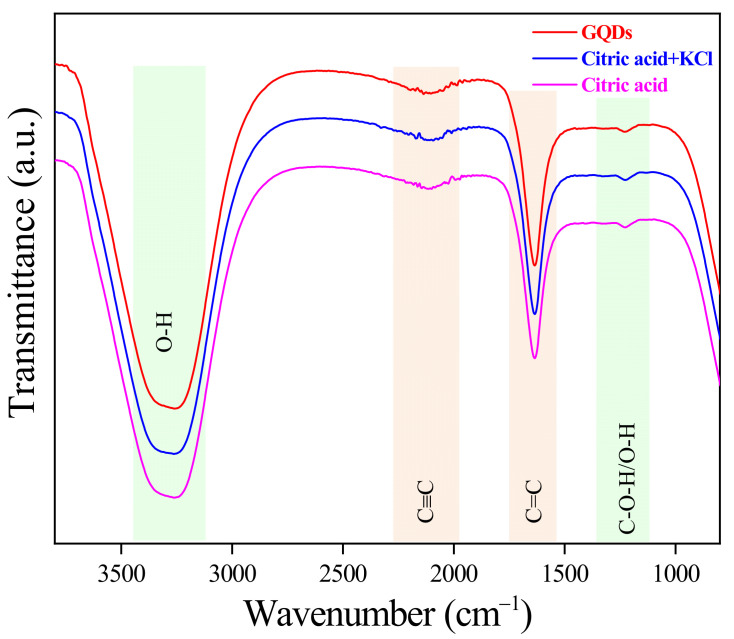
FTIR transmittance spectra of GQDs at different electrolyte solutions.

Since GQDs are synthesized in a solution-based process, the sizes of GQDs are further analyzed via DLS to obtain a hydrodynamic size. The hydrodynamic sizes of GQDs are shown in Figure 3a compared with the TEM size. It can be seen that the hydrodynamic size is larger than that of the TEM size for all conditions. To explain the larger hydrodynamic size, FTIR spectroscopy is used for further investigation. Figure 4 shows FTIR transmittance spectra of GQDs at various electrolytes. The absorption peaks of FTIR are contained of 3261 cm^−1^, 2136 cm^−1^, 1635 cm^−1^, and 1230 cm^−1^ peaks that are assigned and fitted to O-H, C≡C, C=C, and C-O-H/O-H, respectively [20,21]. This FTIR result confirms the presence of COOH^−^ or OH^−^ functional groups on GQDs. This implies the negligible oxygen impurity that grows on GQDs and is in good correspondence with the scissoring model where OH^−^ plays an important role in exfoliating the GQDs from graphite rods [17], inducing the enhancement of the hydrodynamic layer size on GQDs edges. Therefore, the hydroxyl molecules bonding around the GQDs surface, called the hydrodynamic layer form of GQDs, also produced the GQDs larger than GQDs from the TEM images.

The synthesis of GQDs is based on the exfoliation process of the graphite rods with the induction of OH^−^ ions via the oxidation reaction process. This process is produced by the surface charge on GQDs by ions dissolved in the electrolyte solution that become segregated and reoriented upon being dispersed in colloidal forms under the equilibrium of GQDs in the electrical field by the electrochemical process. Therefore, this equilibrium state of GQDs resulted in a significantly larger generated surface charge layer, which is displayed as the hydrodynamic sizes of GQDs depending on various types of ions dispersed in the solution. Considering the citric acid electrolyte, it is the triprotic acid that could be extracted at three protons per one molecule. Therefore, the molecule extraction of citric acid at 0.1M is the minimum required KCl at a concentration of 0.3M to neutralize all of the citric ions [17]. It is also noteworthy that the hydrodynamic size of GQDs grown with KCl 0.3M (29 nm) and 0.45M (27 nm) are relatively smaller than those prepared with other KCl concentrations. This is likely because the citrate ions in the solution are already neutralized by K^+^, allowing smaller ions to gather near the ion cores. Hence, reducing the KCl concentration to 0.15M naturally increased the size of the hydrodynamic layer on GQDs since the remaining citrate ions are required to neutralize K^+^ ions. These ions attracted other OH^−^ ions to form as large molecules on the surface of GQDs, resulting in a large hydrodynamic layer size observed for 127 nm at the 0.15M condition. Furthermore, the larger size of the hydrodynamic layer (69 nm) in Figure 3a was produced by the increasing KCl concentration up to 0.60M, which is caused by the over-saturated Cl^−^ ions being beyond the equilibrium concentration of citric acid. Additionally, these oversaturated Cl^−^ ions induce the deep eutectic solvents phenomenon [22], which increased the rapid mobility of Cl^−^ ions and generated the electric field via the mobility of Cl^−^ ions around the proton that affected the larger hydrodynamic layer.

The information about the hydrodynamic size could be examined as the feature of the stability colloidal in GQDs by the zeta potential technique [23], and the zeta potential of GQDs is shown in Table 1. The negative zeta potential of GQDs at the range of −10 to −50 mV is observed at various electrolyte concentrations, suggesting the stable solutions of GQDs. Furthermore, this information revealed the presence of the surface charge layer, stern layer, slipping plan layer, and ionic shell layer [24], which was formed together with the hydrodynamic size, as represented in the schematic diagram in Figure 3b.

### 2.3. Growth Mechanism of GQDs

**Figure 5:**
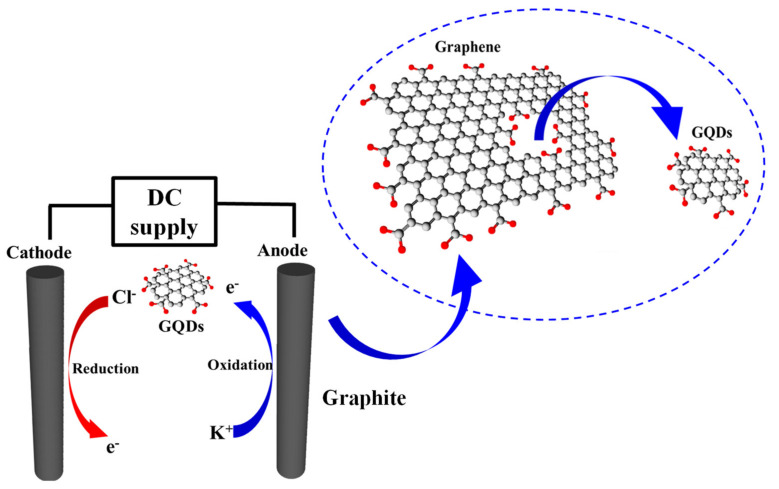
Schematic diagram of GQDs growth mechanism in the electrochemical exfoliation process via graphite rods electrodes.

From the characterization results, the growth mechanism of GQDs can be explained via chemical reactions taking place in Figure 5 as in Equations (1)–(9). In the growth process, citrate acid is dissolved in water under an electric field, providing hydrolysis in water and creating H^+^ and OH^−^ ions in an electrochemical reaction. The citric acid can dissociate three times for H^+^ ions due to its triprotic properties as described in Equations (1)–(3). Therefore, most ions in this solution are OH^−^ ions and can be used as intercalated ions between the layers of graphite rods to exfoliate the C_x_OH molecules via the oxidation reaction of the water at the anode [17] when the electric field is applied. Normally, OH^−^ ions are oxidized at the sites of defects of graphite rods, inducing oxygen being trapped at interface layers of the anode graphite and breaking the van der Waals force of graphite layers by the pressure of the inserted oxygen existing at the defect site to obtain the particle dots as GQDs, as described in Equations (5) and (6). In contrast, the oxygen at the cathode side becomes the oxygen radical by accepting an extra electron in (7). Then H_2_O_2_ is created as described in Equation (8). These OH^−^ and oxygen radicals in (9) are used to peel off graphite layers to obtain the nanoscale exfoliated graphite particles [25].

Regarding GQDs, each condition for electrolyte concentrations of 0.15M to 0.60M is overfilled in giving K^+^ and Cl^−^ according to Equation (4). Due to the electrolyte compound of citric acid and KCl being involved in the deep eutectic solvent (DES) in this solution, which has the combination of hydrogen bond donors and halogenic ions under the low-temperature in liquid forms as nanoparticles, the entropy of the system also increased after the mixing [22]. Influenced by DES on the grown GQDs exhibited by the high solubility of small-sized, dispersed nanoparticles [26] and higher carbon dioxide solubility [27] in exfoliation form [17,25], we also observed the production of QDs similar to OH^−^ ions oxidation into oxygen, peeling the graphite into GQDs [17]. As a result, the significance of OH^−^ ions in the electrochemical process is worth achieving GQDs, as the nanoparticle size is 1.7–2.0 nm for the electrochemical method.

Solution:

(1) $$H_{3}C⇌H^{+}+H_{2}C^{-}$$

(2) $$H_{2}C^{-}⇌H^{+}+HC^{2-}$$

(3) $$HC^{2-}⇌H^{+}+C^{3-}$$

(4) $$KCl\to K^{+}+Cl^{-}$$

Anode:

(5) $$OH^{-}+C_{x}\to C_{x}OH+e^{-}$$

(6) H2O⇌12O2+2H++2e−

Cathode:

(7) $$O_{2}+e^{-}\to \cdot \;O_{2}^{-}$$

(8) $$2H^{+}+\cdot \;O_{2}^{-}+\cdot \;O_{2}^{-}\to H_{2}O_{2}+O_{2}$$

(9) $$H_{2}O_{2}+e^{-}\to OH^{-}+\cdot \;OH$$

### 2.4. Analysis of SERS in GQDs

**Figure 6:**
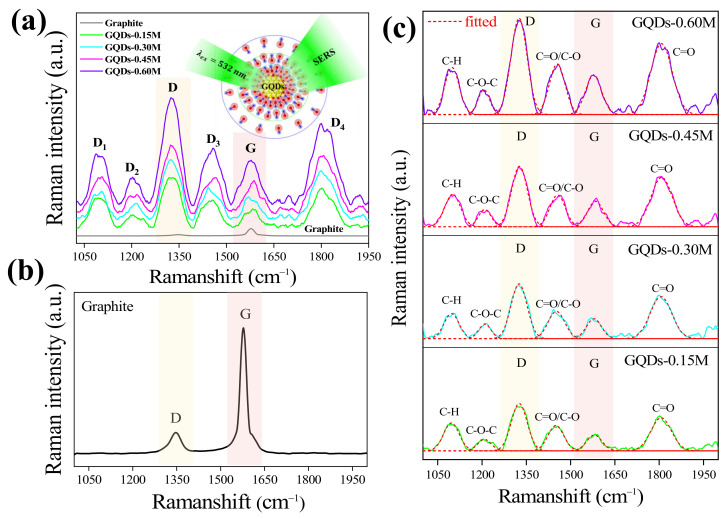
(**a**) Raman spectra of GQDs at various electrolyte concentrations with (**b**) Raman spectrum of the normal graphite for comparison and (**c**) fitted Raman spectra of GQDs with a Gaussian function to obtain the peak positions.

Figure 6a shows Raman spectra of GQDs at various electrolyte concentrations with the Raman spectrum of the normal graphite for comparison as seen in Figure 6b. Interestingly, the Raman intensity of GQDs is much higher than that of normal graphite indicating SERS signals.

The SERS peaks are fitted with a Gaussian function as shown in Figure 6c and the peak positions are summarized in Table 2. The SERS peak positions are at 1096 cm^−1^, 1208 cm^−1^, 1325 cm^−1^, 1450 cm^−1^, 1580 cm^−1^, and 1806 cm^−1^. These peaks can be assigned as D_1_-D_2_, D, D_3_, G, and D_4_ peaks according to carbon-hydrogen bond (C-H), carboxylic group (COOH/C-OH), defect signals of zigzag-armchair on carbon, carbon-oxygen bond (C=O/C-O), carbon double bonds (C=C), and carbon-oxygen double bonds (C=O) [2,28,29], respectively. Moreover, the SERS peak positions are about the same for all conditions, indicating a similar vibrational frequency and similar mechanism.

It should be noted that the SERS on GQDs is also observed at 1326 cm^−1^ for the D peak and at 1584 cm^−1^ for G peaks, similar to the Raman spectrum of the normal graphite, in which the D peak is defined as the presence of impurity defects by the breathing mode on the carbon structure of GQDs and the G peak is the in-plane symmetry on the sp^2^ orbital of the carbon signal [28,29]. Thus, D and G peaks are further analyzed via the Raman enhancement factor (*EF*) by using the Raman intensity ratio of GQDs (*I_GQDs_*) and graphite (*I_graphite_*) [15,16] in each position, expressed as Equation (10) and shown in Figure 6a.

(10) $$EF=\frac{I_{\;GQDs}}{I_{graphite}}$$

**Figure 7:**
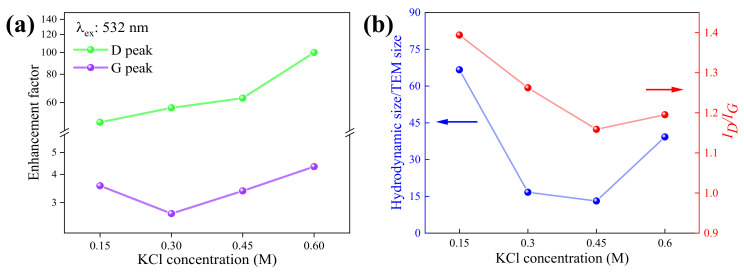
(**a**) Enhancement Factor of D and G peaks at various electrolyte concentrations, and (**b**) *I_D_/I_G_* and the size ratio between the hydrodynamic layer size and TEM size at various concentrations.

For SERS analysis of GQDs, the Raman intensity at D and G peaks are used to determine *EF* and *EF* at various electrolyte concentrations, as plotted in Figure 7a. It can be seen that the highest *EF* on GQDs is obtained at the D position peak with an *EF* of up to 100 for GQDs-0.60M and 49 for GQDs-0.15M. In contrast, the G peak of GQDs is shown with an *EF* in the range of 3.6 to 4.3, which is much lower than that of the D peak. Since the D peak is related to the impurity defect level, the enhancement of Raman intensity is definitely related to the defect of GQDs [28] such as hydroxyl molecules bonded (OH^−^ and COOH^−^) with GQDs edges [28,29,30,31]. The origin of defects state in GQDs are typically caused by the functional groups (C-OH, COOH), attached at the edges of GQDs such as zigzag and armchair edges site [30,32]. The exfoliation process via electrochemical synthesis in GQDs enables the presence of defect states by C-OH and COOH functional groups on surface GQDs. Moreover, the redshift of the D peak compared to the normal graphite can be observed, suggesting the presence of strong defects on GQDs [31].

To discuss the number of impurity defects on GQDs, the ratio of D and G peaks’ intensity (*I_D_/I_G_*) of GQDs Raman spectra [28] are assigned to consider or the defect evaluation on GQDs, as shown in Figure 7b. As a result, *I_D_/I_G_* on GQDs in electrolyte concentrations with 0.15M to 0.45M are reduced, which exhibited the recovery of the GQDs defect while the concentration increased. Then, this *I_D_/I_G_* ratio becomes stable by GQDs with 0.45M and 0.60M Furthermore, GQDs are dispersed in an aqueous solution as colloidal forms, which included hydroxyl molecules (OH^−^) and hydrocarbon (C-H), that had generally formed at GQDs edges and might induce defects on the GQDs edges [30]. However, the defects caused by hydroxyl molecules on GQDs edges induced either in-plane or out-plane defects, resulting in significantly different hydrodynamic sizes and TEM sizes, which is represented by the ratio versus electrolyte concentrations as shown in Figure 7b. The ratios of the hydrodynamic size and the TEM size of GQDs represent a similar trend to the amount of impurity defect on GQDs under the change of electrolyte concentrations. Therefore, the hydroxyl molecules-induced defect at GQDs edges can be correlated to the hydrodynamic size and TEM sizes.

Besides, the obtained SERS signals can be explained by the enhancement of the CM mode, which is similar to the previously reported work [15]. In addition, analysis of SERS in GQDs with Rhodamine B (RhB) is demonstrated for comparison in Supplementary Materials Figure S1.

## 3. Materials and Methods

GQDs were prepared by the electrochemical method with graphite rods as electrodes. The graphite rods with 5 mm diameter and 5 cm length were obtained from pencil cores (STAEDTLER Mars Lumograph (EAN 40 07817 100295) Art. Nt. 100EE) and were cleaned in acetone via sonication for 5 min following by annealing at 450 °C for 15 min similar to the previous work [17]. Next, an electrolyte solution (50 mL) was prepared by mixing critic acid 0.1M (99.5%) with KCl (99.8%) at various concentrations of 0.15M, 0.30M, 0.45Mm, and 0.60M in deionizing water (DI water). The electrochemical process is driven by a DC supply for 4–10 V of voltage (Agilent E3633A) across the electrodes. The bias voltage was firstly ramped up to 4 V over the first hour to pre-treat the graphite surface and then kept constant at 10 V for another 4 h during the growth process. After the process, the obtained GQDs solutions were filtered twice with a 0.45-µm of size filter before further analysis.

GQDs were characterized by determining the particle size of GQDs by dynamic light scattering (DLS), collecting the morphology picture of GQDs via TEM spectroscopy (JEOL JEM-2010), testing the stability of GQDs by Zeta potential (Horiba SZ-100), and identifying the chemical bonding on GQDs via FTIR. The survey scan of SERS in GQDs was measured by Raman spectroscopy (XploRA Horiba KH8700) with a 532 nm excitation Raman source. The laser beam was focused by a 50× objective lens, resulting in a spot size of around 2 μm in diameter. The acquisition time was 4.5 s for each spectrum.

## 4. Conclusions

GQDs are successfully grown via the electrochemical process with citric acid and KCl electrolyte solution to obtain GQDs in colloidal solution-based format. The typical GQDs particles are in a spherical shape for sizes of 1.7 to 2.0 nm. The FTIR results confirm the presence of COOH^−^ or OH^−^ functional groups on GQDs, leading to a larger hydrodynamic size. SERS signals are observed via GQDs spectra through the Raman spectra at D (1326 cm^−1^) and G (1584 cm^−1^). The increasing concentration of KCl in the electrolyte solution for 0.15M to 0.60M demonstrated the increment of Raman intensity at the D peak of GQDs up to 100 over the D peak of graphite. This result reveals the potential feasibility of GQDs as SERS applications.

## Figures and Tables

**Table 1:** Comparison of hydrodynamic size and zeta potential at various KCl concentrations.

KCl Concentration (M)	Hydrodynamic Size (nm)	Zeta Potential (mV)
GQDs-0.15M	127 ± 23	−36 ± 7
GQDs-0.30M	29 ± 6	−50 ± 14
GQDs-0.45M	27 ± 6	−15 ± 17
GQDs-0.60M	69 ± 13	−39 ± 12

**Table 2:** Summary of the fitted Raman peak position in the region 1050–1950 cm^−1^. The peak identity is also assigned to each Raman peak position.

Sample	Fitted Raman Peak Position (cm^−1^)					
	D_1_	D_2_	D	D_3_	G	D_4_
Graphite	-	-	1343	-	1575	-
GQDs-0.15M	1096	1208	1326	1450	1581	1807
GQDs-0.30M	1097	1210	1325	1452	1579	1806
GQDs-0.45M	1101	1209	1327	1456	1584	1808
GQDs-0.60M	1098	1206	1325	1455	1578	1808
Peak identity	sp^2^–sp^3^carbon/ C-H bending	C-O-C	Zigzag/armchair Edge states	C=O/C-O	C=C	C=O stretch

## Data Availability

Data sharing not applicable. No new data were created or analyzed in this study.

## Supplementary Materials

The following are available online. Figure S1: SERS spectra of GQDs compared to SERS spectra of RhB for 10^−9^ M based on GQDs.

## Sample Availability

Samples of the compounds are not available from the authors.
