# Supplementary material for: Surface Enhanced Raman Scattering in Graphene Quantum Dots Grown via Electrochemical Process
Source: Molecules. 2021 Sep 9;26(18):5484. doi: 10.3390/molecules26185484 (PMC8471654; doi:10.3390/molecules26185484)
Supplement: Supplementary file 1 [file molecules-26-05484-s001.zip › molecules-1348432-supplementary.pdf]

Supplementary Material

Surface Enhanced Raman Scattering in Graphene Quantum Dots Grown via Electrochemical Process

Rangsan Panyathip [1], Sukrit Sucharitakul [1], Surachet Phaduangdhitidhada [1], Athipong Ngamjarurojana [1], Pisist Kumnorkaew [2] and Supab Choopun [1,*]

Analysis of SERS in GQD with Rhodamine B (RhB)

The SERS effects on GQDs substrates are measured with 532 nm laser excitation in the presence of 10^{-9} M RhB as shown in Figure S1. Interestingly, a significant enhancement in Raman peaks of RhB at 1326 cm^{-1}, and 1571 cm^{-1} or D and G peaks are observed on the GQDs substrate.

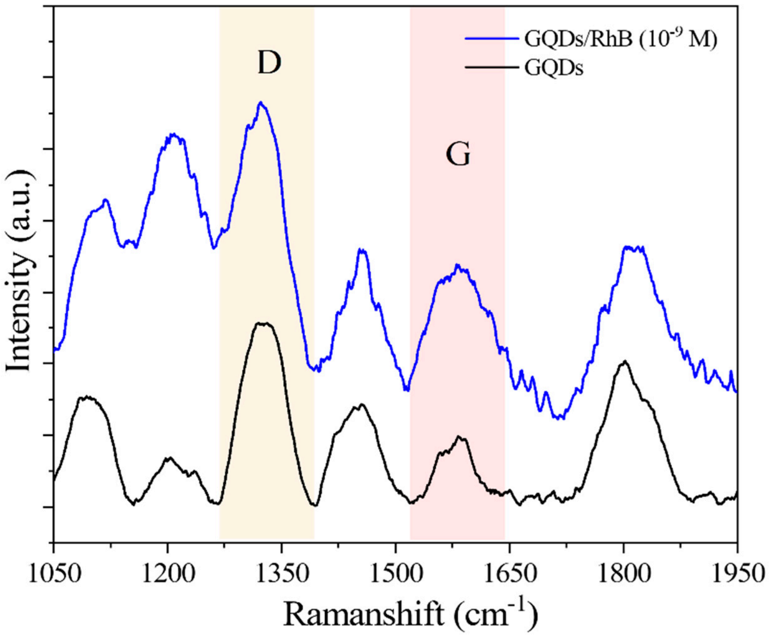

Figure S1. SERS spectra of GQDs compared to SERS spectra of RhB for 10^{-9} M based on GQDs.

Besides, the *EF* of SERS spectra are calculated by D and G peaks intensity of GQDs compared with RhB treated on the GQDs substrate. Experimentally, the *EF* is defined in Equation (S1) [16]. As the SERS process depends on the effective number of adsorbed molecules, Equation (S1) is modified as:

$$EF = \frac{I_{SERS}}{I_R} \times \frac{M_R}{M_{SERS}} \tag{S1}$$

where I_{SERS} and I_R are the Raman intensity at D and G of GQDs with and without the presence of RhB at the known concentration, respectively. M_R (10^{-7}) is the concentration of RhB molecules contributing to the bulk Raman signal and M_{SERS} (10^{-9}) is the concentration of RhB-absorbed molecules that indicated a SERS Raman signal. Hence, the value of EF on GQDs is presented as about 2.38×10^3 and 8.90×10^2 at D and G peaks with an RhB concentration of 10^{-9}M.

16. Das, R.; Parveen, S.; Bora, A.; Giri, P.K. Origin of high photoluminescence yield and high SERS sensitivity of nitrogen-doped graphene quantum dots. Carbon 2020, 160, 273–286, doi:10.1016/j.carbon.2020.01.030.
